# 1-(2,6-Dichloro­benzo­yl)-3-(3-nitro­phen­yl)thio­urea dimethyl­formamide solvate

**DOI:** 10.1107/S1600536809055056

**Published:** 2010-01-09

**Authors:** Min Li, Dongxiao Hou

**Affiliations:** a Department of Chemistry, Changzhi University, Shanxi, People’s Republic of China; b Institute of Applied Chemistry, Shanxi University, Shanxi, People’s Republic of China

## Abstract

In the title compound, C_14_H_9_Cl_2_N_3_O_3_S·C_3_H_7_NO, the two aromatic rings enclose a dihedral angle of 32.93 (12)°. The thiourea mol­ecule exists in its thione form in the solid state with typical C=S and C—N bond lengths.  In the crystal, N—H⋯O hydrogen bonds exist between the thio­urea and carbonyl groups on the same and neighboring mol­ecules. In addition, each dimethyl­formamide solvate mol­ecule forms a hydrogen bond to one N atom of the thio­urea group.

## Related literature

For general background to the use of thio­urea and urea derivatives in the development of agrochemicals and pharmacological agents, see: Darlington *et al.* (1996 ▶); Dowding & Leeds (1971 ▶); Sasse *et al.* (1969 ▶). For bond lengths in other other substituted thio­ureas, see: Khawar Rauf *et al.* (2006*a* ▶,**b* ▶,c* ▶, 2007 ▶, 2009 ▶). For previously reported C=S distances, see: Bailey *et al.* (1997 ▶).
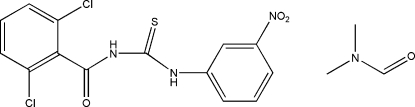


## Experimental

### 

#### Crystal data

C_14_H_9_Cl_2_N_3_O_3_S·C_3_H_7_NO
*M* *_r_* = 443.30
Triclinic, 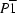

*a* = 8.507 (5) Å
*b* = 10.240 (5) Å
*c* = 12.414 (8) Å
α = 70.40 (4)°
β = 81.74 (5)°
γ = 87.98 (4)°
*V* = 1008 (1) Å^3^
*Z* = 2
Mo *K*α radiation
μ = 0.46 mm^−1^
*T* = 293 K
0.30 × 0.20 × 0.20 mm

#### Data collection

Bruker SMART APEX CCD area-detector diffractometer
Absorption correction: multi-scan (*SADABS*; Sheldrick, 1997 ▶) *T*_min_ = 0.875, *T*_max_ = 0.914
4551 measured reflections
3413 independent reflections
2691 reflections with *I* > 2σ(*I*)
*R*_int_ = 0.021

#### Refinement

*R*[*F* ^2^ > 2σ(*F* ^2^)] = 0.046
*wR*(*F* ^2^) = 0.139
*S* = 1.11
3413 reflections
256 parameters
H-atom parameters constrained
Δρ_max_ = 0.30 e Å^−3^
Δρ_min_ = −0.42 e Å^−3^

### 

Data collection: *SMART* (Siemens, 1996 ▶); cell refinement: *SAINT* (Siemens, 1996 ▶); data reduction: *SAINT*; program(s) used to solve structure: *SHELXS97* (Sheldrick, 2008 ▶); program(s) used to refine structure: *SHELXL97* (Sheldrick, 2008 ▶); molecular graphics: *ORTEP-3 for Windows* (Farrugia, 1997 ▶); software used to prepare material for publication: *SHELXL97*.

## Supplementary Material

Crystal structure: contains datablocks global, I. DOI: 10.1107/S1600536809055056/ez2196sup1.cif

Structure factors: contains datablocks I. DOI: 10.1107/S1600536809055056/ez2196Isup2.hkl

Additional supplementary materials:  crystallographic information; 3D view; checkCIF report

## Figures and Tables

**Table 1:** Hydrogen-bond geometry (Å, °)

*D*—H⋯*A*	*D*—H	H⋯*A*	*D*⋯*A*	*D*—H⋯*A*
N2—H2⋯O1^i^	0.86	2.47	3.182 (3)	141
N2—H2⋯O1	0.86	1.99	2.675 (3)	136
N1—H1⋯O4^ii^	0.86	1.96	2.787 (3)	161

Symmetry codes: (i) 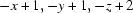; (ii) 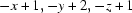.

**Fig. 1.**
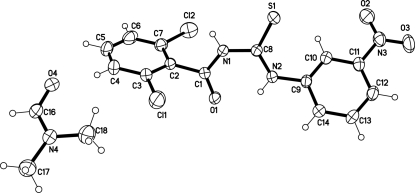
View of a molecule of (I) showing the atom-labelling scheme. Displacement ellipsoids are drawn at the 50% probability level. H atoms are omitted for clarity.

**Fig. 2.**
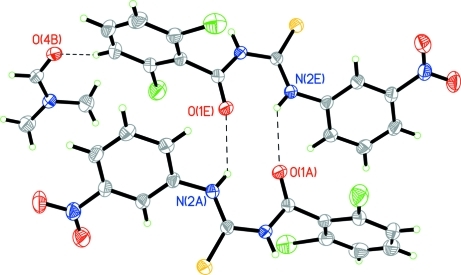
Partial packing view of (I). Displacement ellipsoids are drawn at the 30% probability level. Hydrogen bonds are shown as dashed lines.

## supplementary crystallographic information

### Comment

Earlier studies have shown that thiourea and urea derivatives have played an important role in developing agrochemicals and pharmacological agents (Dowding & Leeds, 1971; Sasse *et al.*, 1969; Darlington *et al.*, 1996). As part of our interest in N,*N*'-disubstituted thioureas, we now report the crystal structure of the title compound (I).

The N—C bonds in (I), see Fig. 1, differ significantly from one another but are short in comparison with the typical value for an N—C single bond (1.479 Å). Owing to the introduction of the C=O electron-acceptor group the adjacent C-S bond length [1.652 (2)Å ] is shorter than previously reported C=S distances (1.710 (7)Å) (Bailey, *et al.*, 1997). These distances are similar to those usually found in other substituted thioureas (Khawar Rauf *et al.*, 2006*a*, 2006*b*, 2006*c*, 2007, 2009). The dihedral angle between the aromatic rings is 32.93 (12)°, and the corresponding angles with the thiourea plane are 83.52 (7)° for the C2–C7 ring and 50.61 (7)° for the C9–C14 ring.The thiocarbonyl and carbonyl groups are almost coplanar.

Inter- and intramolecular N—H···O hydrogen bonds exist between the thiourea N—H-atoms and carbonyl-O atoms. In addition, each dimethylformamide solvate molecule also has a hydrogen bond to the N of the thiourea groups (2.787 (3) Å: Table 1, Fig. 2).

### Experimental

Freshly prepared 2,6-dichlorobenzoylisothiocyanate (2.32 g, 10 mmol) was added to dimethylformamide (30 ml) and stirred for 2 minutes. Afterwards neat 3-nitroaniline (1.38 g, 10 mmol) was added and the resulting mixture was stirred for 1 h.The reaction mixture was then poured into an ice-water mixture and stirred well. The solid product was separated and washed with deionized water and purified by recrystallization from methanol/CH_2_Cl_2_ (1:1 *v*/*v*) to give fine crystals of the title compound (I), with an overall yield of 85%.

### Refinement

All H atoms were positioned geometrically, with C—H = 0.96– 0.98 Å, and refined as riding, allowing for free rotation of the methyl groups. The *U*_iso_(H) values were set at 1.5*U*_eq_(C).

### Crystal data

**Table:**

C_14_H_9_Cl_2_N_3_O_3_S·C_3_H_7_NO	*Z* = 2
*M**_r_* = 443.30	*F*(000) = 456
Triclinic, *P*1	*D*_x_ = 1.460 Mg m^−^^3^
*a* = 8.507 (5) Å	Mo *K*α radiation, λ = 0.71073 Å
*b* = 10.240 (5) Å	Cell parameters from 1797 reflections
*c* = 12.414 (8) Å	θ = 2.4–25.9°
α = 70.40 (4)°	µ = 0.46 mm^−^^1^
β = 81.74 (5)°	*T* = 293 K
γ = 87.98 (4)°	Block, yellow
*V* = 1008 (1) Å^3^	0.30 × 0.20 × 0.20 mm

### Data collection

**Table:**

Bruker SMART APEX CCD area-detector diffractometer	3413 independent reflections
Radiation source: fine-focus sealed tube	2691 reflections with *I* > 2σ(*I*)
graphite	*R*_int_ = 0.021
phi and ω scans	θ_max_ = 25.0°, θ_min_ = 2.4°
Absorption correction: multi-scan (*SADABS*; Sheldrick, 1997)	*h* = −10→9
*T*_min_ = 0.875, *T*_max_ = 0.914	*k* = −12→12
4551 measured reflections	*l* = −14→13

### Refinement

**Table:**

Refinement on *F*^2^	Secondary atom site location: difference Fourier map
Least-squares matrix: full	Hydrogen site location: inferred from neighbouring sites
*R*[*F*^2^ > 2σ(*F*^2^)] = 0.046	H-atom parameters constrained
*wR*(*F*^2^) = 0.139	*w* = 1/[σ^2^(*F*_o_^2^) + (0.0842*P*)^2^ + 0.0267*P*] where *P* = (*F*_o_^2^ + 2*F*_c_^2^)/3
*S* = 1.11	(Δ/σ)_max_ = 0.001
3413 reflections	Δρ_max_ = 0.30 e Å^−^^3^
256 parameters	Δρ_min_ = −0.42 e Å^−^^3^
0 restraints	Extinction correction: *SHELXL97* (Sheldrick, 2008), Fc^*^=kFc[1+0.001xFc^2^λ^3^/sin(2θ)]^-1/4^
Primary atom site location: structure-invariant direct methods	Extinction coefficient: 0.082 (8)

### Special details

**Table:**

Geometry. All e.s.d.'s (except the e.s.d. in the dihedral angle between two l.s. planes) are estimated using the full covariance matrix. The cell e.s.d.'s are taken into account individually in the estimation of e.s.d.'s in distances, angles and torsion angles; correlations between e.s.d.'s in cell parameters are only used when they are defined by crystal symmetry. An approximate (isotropic) treatment of cell e.s.d.'s is used for estimating e.s.d.'s involving l.s. planes.
Refinement. Refinement of *F*^2^ against ALL reflections. The weighted *R*-factor *wR* and goodness of fit *S* are based on *F*^2^, conventional *R*-factors *R* are based on *F*, with *F* set to zero for negative *F*^2^. The threshold expression of *F*^2^ > σ(*F*^2^) is used only for calculating *R*-factors(gt) *etc*. and is not relevant to the choice of reflections for refinement. *R*-factors based on *F*^2^ are statistically about twice as large as those based on *F*, and *R*- factors based on ALL data will be even larger.

### Fractional atomic coordinates and isotropic or equivalent isotropic displacement parameters (Å^2^)

**Table:**

	*x*	*y*	*z*	*U*_iso_*/*U*_eq_	
S1	0.05945 (7)	0.84634 (6)	0.96546 (5)	0.0570 (3)	
Cl1	0.47677 (12)	0.70890 (8)	0.61611 (6)	0.0911 (3)	
Cl2	0.67130 (10)	0.96356 (8)	0.88967 (7)	0.0831 (3)	
N1	0.3397 (2)	0.80330 (17)	0.86720 (15)	0.0445 (4)	
H1	0.3226	0.8832	0.8187	0.053*	
N2	0.2412 (2)	0.62210 (17)	1.03144 (15)	0.0481 (5)	
H2	0.3272	0.5811	1.0161	0.058*	
N3	−0.0870 (3)	0.6004 (2)	1.39465 (16)	0.0594 (5)	
O1	0.52143 (19)	0.63011 (15)	0.90012 (14)	0.0577 (5)	
O2	−0.0391 (3)	0.7149 (2)	1.38288 (17)	0.0829 (6)	
O3	−0.1862 (2)	0.5355 (2)	1.47485 (16)	0.0832 (6)	
C1	0.4823 (2)	0.7462 (2)	0.84468 (18)	0.0433 (5)	
C2	0.5907 (3)	0.8398 (2)	0.74414 (18)	0.0466 (5)	
C3	0.6004 (3)	0.8284 (2)	0.6353 (2)	0.0588 (6)	
C4	0.7002 (4)	0.9109 (3)	0.5417 (2)	0.0786 (9)	
H4	0.7032	0.9025	0.4692	0.094*	
C5	0.7950 (4)	1.0058 (3)	0.5583 (3)	0.0857 (10)	
H5	0.8647	1.0609	0.4962	0.103*	
C6	0.7900 (3)	1.0218 (3)	0.6639 (3)	0.0788 (9)	
H6	0.8553	1.0868	0.6734	0.095*	
C7	0.6859 (3)	0.9394 (2)	0.7564 (2)	0.0569 (6)	
C8	0.2176 (3)	0.7497 (2)	0.95824 (18)	0.0426 (5)	
C9	0.1355 (2)	0.5494 (2)	1.13216 (18)	0.0444 (5)	
C10	0.0778 (3)	0.6112 (2)	1.21291 (18)	0.0477 (5)	
H10	0.1048	0.7024	1.2021	0.057*	
C11	−0.0212 (3)	0.5334 (2)	1.31033 (18)	0.0496 (5)	
C12	−0.0602 (3)	0.3960 (2)	1.3312 (2)	0.0579 (6)	
H12	−0.1252	0.3452	1.3981	0.070*	
C13	0.0003 (3)	0.3373 (2)	1.2498 (2)	0.0624 (7)	
H13	−0.0241	0.2452	1.2617	0.075*	
C14	0.0970 (3)	0.4129 (2)	1.1505 (2)	0.0536 (6)	
H14	0.1364	0.3718	1.0958	0.064*	
N4	0.6148 (2)	0.7230 (2)	0.23093 (16)	0.0561 (5)	
O4	0.6593 (2)	0.91813 (16)	0.26969 (14)	0.0645 (5)	
C16	0.6896 (3)	0.8409 (2)	0.2126 (2)	0.0549 (6)	
H16	0.7720	0.8675	0.1514	0.066*	
C17	0.6543 (4)	0.6418 (3)	0.1547 (2)	0.0737 (8)	
H17A	0.7418	0.6852	0.0969	0.110*	
H17B	0.5639	0.6365	0.1181	0.110*	
H17C	0.6834	0.5500	0.1989	0.110*	
C18	0.4832 (4)	0.6777 (3)	0.3233 (3)	0.0856 (9)	
H18A	0.4517	0.7523	0.3519	0.128*	
H18B	0.5151	0.6003	0.3847	0.128*	
H18C	0.3954	0.6503	0.2946	0.128*	

### Atomic displacement parameters (Å^2^)

**Table:**

	*U*^11^	*U*^22^	*U*^33^	*U*^12^	*U*^13^	*U*^23^
S1	0.0485 (4)	0.0507 (4)	0.0612 (4)	0.0082 (3)	0.0081 (3)	−0.0122 (3)
Cl1	0.1307 (8)	0.0806 (5)	0.0656 (5)	−0.0230 (5)	−0.0006 (4)	−0.0318 (4)
Cl2	0.0883 (6)	0.0810 (5)	0.0918 (6)	−0.0026 (4)	−0.0312 (4)	−0.0362 (4)
N1	0.0443 (10)	0.0372 (8)	0.0438 (10)	0.0025 (7)	0.0030 (8)	−0.0068 (7)
N2	0.0432 (10)	0.0420 (9)	0.0505 (11)	0.0018 (8)	0.0079 (8)	−0.0104 (8)
N3	0.0614 (13)	0.0695 (14)	0.0425 (11)	0.0034 (11)	−0.0032 (10)	−0.0143 (10)
O1	0.0545 (9)	0.0450 (8)	0.0562 (9)	0.0103 (7)	0.0075 (7)	−0.0012 (7)
O2	0.1064 (16)	0.0775 (13)	0.0678 (12)	−0.0047 (12)	0.0060 (11)	−0.0351 (10)
O3	0.0753 (13)	0.1017 (15)	0.0586 (11)	−0.0029 (11)	0.0196 (10)	−0.0198 (11)
C1	0.0445 (12)	0.0407 (11)	0.0425 (11)	0.0002 (9)	−0.0002 (9)	−0.0132 (9)
C2	0.0427 (11)	0.0406 (11)	0.0476 (12)	0.0038 (9)	0.0040 (9)	−0.0078 (9)
C3	0.0645 (15)	0.0500 (13)	0.0525 (14)	0.0020 (11)	0.0107 (11)	−0.0128 (11)
C4	0.090 (2)	0.0636 (16)	0.0582 (16)	0.0079 (16)	0.0252 (15)	−0.0043 (13)
C5	0.073 (2)	0.0604 (17)	0.089 (2)	0.0000 (15)	0.0355 (17)	0.0011 (16)
C6	0.0540 (16)	0.0512 (14)	0.113 (3)	−0.0089 (12)	0.0091 (16)	−0.0105 (15)
C7	0.0453 (13)	0.0490 (12)	0.0693 (16)	−0.0002 (10)	−0.0003 (11)	−0.0136 (11)
C8	0.0456 (12)	0.0386 (10)	0.0419 (11)	−0.0041 (9)	0.0010 (9)	−0.0139 (9)
C9	0.0387 (11)	0.0455 (11)	0.0425 (11)	−0.0026 (9)	0.0003 (9)	−0.0082 (9)
C10	0.0470 (12)	0.0460 (11)	0.0469 (12)	−0.0045 (9)	−0.0015 (10)	−0.0126 (10)
C11	0.0458 (12)	0.0577 (13)	0.0407 (12)	−0.0010 (10)	−0.0015 (10)	−0.0119 (10)
C12	0.0570 (14)	0.0557 (13)	0.0482 (13)	−0.0066 (11)	0.0026 (11)	−0.0036 (11)
C13	0.0654 (16)	0.0449 (12)	0.0651 (15)	−0.0113 (11)	0.0052 (13)	−0.0076 (11)
C14	0.0552 (14)	0.0456 (12)	0.0558 (14)	−0.0004 (10)	0.0015 (11)	−0.0153 (10)
N4	0.0623 (12)	0.0489 (11)	0.0509 (11)	−0.0033 (9)	−0.0015 (9)	−0.0110 (9)
O4	0.0862 (13)	0.0446 (8)	0.0560 (10)	0.0051 (8)	−0.0006 (9)	−0.0123 (8)
C16	0.0620 (15)	0.0470 (12)	0.0465 (13)	0.0028 (11)	−0.0039 (11)	−0.0052 (10)
C17	0.094 (2)	0.0662 (16)	0.0650 (17)	−0.0082 (15)	−0.0123 (15)	−0.0254 (13)
C18	0.077 (2)	0.0725 (18)	0.096 (2)	−0.0153 (15)	0.0155 (17)	−0.0218 (16)

### Geometric parameters (Å, °)

**Table:**

S1—C8	1.652 (2)	C6—H6	0.9300
Cl1—C3	1.738 (3)	C9—C10	1.381 (3)
Cl2—C7	1.740 (3)	C9—C14	1.383 (3)
N1—C1	1.362 (3)	C10—C11	1.384 (3)
N1—C8	1.394 (3)	C10—H10	0.9300
N1—H1	0.8600	C11—C12	1.386 (3)
N2—C8	1.345 (3)	C12—C13	1.373 (4)
N2—C9	1.423 (3)	C12—H12	0.9300
N2—H2	0.8600	C13—C14	1.382 (3)
N3—O2	1.210 (3)	C13—H13	0.9300
N3—O3	1.225 (3)	C14—H14	0.9300
N3—C11	1.472 (3)	N4—C16	1.319 (3)
O1—C1	1.219 (3)	N4—C18	1.448 (4)
C1—C2	1.502 (3)	N4—C17	1.455 (3)
C2—C7	1.384 (3)	O4—C16	1.226 (3)
C2—C3	1.385 (3)	C16—H16	0.9300
C3—C4	1.376 (4)	C17—H17A	0.9600
C4—C5	1.369 (5)	C17—H17B	0.9600
C4—H4	0.9300	C17—H17C	0.9600
C5—C6	1.368 (5)	C18—H18A	0.9600
C5—H5	0.9300	C18—H18B	0.9600
C6—C7	1.387 (4)	C18—H18C	0.9600
			
C1—N1—C8	128.75 (17)	C14—C9—N2	118.5 (2)
C1—N1—H1	115.6	C9—C10—C11	118.0 (2)
C8—N1—H1	115.6	C9—C10—H10	121.0
C8—N2—C9	125.37 (18)	C11—C10—H10	121.0
C8—N2—H2	117.3	C10—C11—C12	122.7 (2)
C9—N2—H2	117.3	C10—C11—N3	118.1 (2)
O2—N3—O3	123.3 (2)	C12—C11—N3	119.2 (2)
O2—N3—C11	118.9 (2)	C13—C12—C11	117.8 (2)
O3—N3—C11	117.8 (2)	C13—C12—H12	121.1
O1—C1—N1	124.13 (19)	C11—C12—H12	121.1
O1—C1—C2	121.96 (19)	C12—C13—C14	121.0 (2)
N1—C1—C2	113.91 (18)	C12—C13—H13	119.5
C7—C2—C3	117.4 (2)	C14—C13—H13	119.5
C7—C2—C1	121.7 (2)	C13—C14—C9	120.1 (2)
C3—C2—C1	121.0 (2)	C13—C14—H14	119.9
C4—C3—C2	122.5 (3)	C9—C14—H14	119.9
C4—C3—Cl1	119.1 (2)	C16—N4—C18	119.9 (2)
C2—C3—Cl1	118.37 (18)	C16—N4—C17	121.3 (2)
C5—C4—C3	118.2 (3)	C18—N4—C17	118.7 (2)
C5—C4—H4	120.9	O4—C16—N4	125.3 (2)
C3—C4—H4	120.9	O4—C16—H16	117.3
C6—C5—C4	121.8 (3)	N4—C16—H16	117.3
C6—C5—H5	119.1	N4—C17—H17A	109.5
C4—C5—H5	119.1	N4—C17—H17B	109.5
C5—C6—C7	119.0 (3)	H17A—C17—H17B	109.5
C5—C6—H6	120.5	N4—C17—H17C	109.5
C7—C6—H6	120.5	H17A—C17—H17C	109.5
C2—C7—C6	121.2 (3)	H17B—C17—H17C	109.5
C2—C7—Cl2	119.17 (18)	N4—C18—H18A	109.5
C6—C7—Cl2	119.6 (2)	N4—C18—H18B	109.5
N2—C8—N1	115.74 (18)	H18A—C18—H18B	109.5
N2—C8—S1	126.52 (17)	N4—C18—H18C	109.5
N1—C8—S1	117.74 (15)	H18A—C18—H18C	109.5
C10—C9—C14	120.3 (2)	H18B—C18—H18C	109.5
C10—C9—N2	121.05 (19)		
			
C8—N1—C1—O1	4.9 (4)	C9—N2—C8—S1	−2.9 (3)
C8—N1—C1—C2	−174.8 (2)	C1—N1—C8—N2	−2.8 (3)
O1—C1—C2—C7	−96.1 (3)	C1—N1—C8—S1	177.82 (18)
N1—C1—C2—C7	83.7 (3)	C8—N2—C9—C10	−50.6 (3)
O1—C1—C2—C3	83.2 (3)	C8—N2—C9—C14	132.3 (2)
N1—C1—C2—C3	−97.1 (2)	C14—C9—C10—C11	−1.3 (3)
C7—C2—C3—C4	0.3 (3)	N2—C9—C10—C11	−178.34 (19)
C1—C2—C3—C4	−178.9 (2)	C9—C10—C11—C12	2.0 (3)
C7—C2—C3—Cl1	−177.59 (18)	C9—C10—C11—N3	−178.0 (2)
C1—C2—C3—Cl1	3.2 (3)	O2—N3—C11—C10	−8.1 (3)
C2—C3—C4—C5	1.2 (4)	O3—N3—C11—C10	173.1 (2)
Cl1—C3—C4—C5	179.1 (2)	O2—N3—C11—C12	171.9 (2)
C3—C4—C5—C6	−1.4 (4)	O3—N3—C11—C12	−6.9 (3)
C4—C5—C6—C7	0.0 (4)	C10—C11—C12—C13	−1.4 (4)
C3—C2—C7—C6	−1.7 (3)	N3—C11—C12—C13	178.7 (2)
C1—C2—C7—C6	177.5 (2)	C11—C12—C13—C14	0.1 (4)
C3—C2—C7—Cl2	176.95 (17)	C12—C13—C14—C9	0.6 (4)
C1—C2—C7—Cl2	−3.8 (3)	C10—C9—C14—C13	0.1 (4)
C5—C6—C7—C2	1.6 (4)	N2—C9—C14—C13	177.2 (2)
C5—C6—C7—Cl2	−177.1 (2)	C18—N4—C16—O4	1.4 (4)
C9—N2—C8—N1	177.71 (19)	C17—N4—C16—O4	176.9 (2)

### Hydrogen-bond geometry (Å, °)

**Table:**

*D*—H···*A*	*D*—H	H···*A*	*D*···*A*	*D*—H···*A*
N2—H2···O1^i^	0.86	2.47	3.182 (3)	141
N2—H2···O1	0.86	1.99	2.675 (3)	136
N1—H1···O4^ii^	0.86	1.96	2.787 (3)	161

Symmetry codes: (i) −*x*+1, −*y*+1, −*z*+2; (ii) −*x*+1, −*y*+2, −*z*+1.
